# Linoleic acid suppresses colorectal cancer cell growth by inducing oxidant stress and mitochondrial dysfunction

**DOI:** 10.1186/1476-511X-9-106

**Published:** 2010-09-24

**Authors:** Xiaofeng Lu, Haining Yu, Qi Ma, Shengrong Shen, Undurti N Das

**Affiliations:** 1 Department of Food Science and Nutrition, College of Biosystems Engineering and Food Science, Zhejiang University, Hangzhou 310029, P R China; 2 College of Pharmaceutical Sciences, Zhejiang University of Technology, Hangzhou 310032, P R China; 3 UND Life Sciences, 13800 Fairhill Road, #321, Shaker Heights, OH 44120, USA; 4 Jawaharlal Nehru Technological University, Kakinada-533 003, India; 5 Krishna Institute of Medical Sciences, Secunderabad-500 003, India

## Abstract

Some polyunsaturated fatty acids (PUFAs), if not all, have been shown to have tumoricidal action, but their exact mechanism(s) of action is not clear. In the present study, we observed that n-6 PUFA linoleic acid (LA) inhibited tumor cell growth at high concentrations (above 300 μM); while low concentrations (100-200 μM) promoted proliferation. Analysis of cell mitochondrial membrane potential, reactive oxygen species (ROS) formation, malondialdehyde (MDA) accumulation and superoxide dismutase (SOD) activity suggested that anti-cancer action of LA is due to enhanced ROS generation and decreased cell anti-oxidant capacity that resulted in mitochondrial damage. Of the three cell lines tested, semi-differentiated colorectal cancer cells RKO were most sensitive to the cytotoxic action of LA, followed by undifferentiated colorectal cancer cell line (LOVO) while the normal human umbilical vein endothelial cells (HUVEC) were the most resistant (the degree of sensitivity to LA is as follows: RKO > LOVO > HUVEC). LA induced cell death was primed by mitochondrial apoptotic pathway. Pre-incubation of cancer cells with 100 μM LA for 24 hr enhanced sensitivity of differentiated and semi-differentiated cells to the subsequent exposure to LA. The relative resistance of LOVO cells to the cytotoxic action of LA is due to a reduction in the activation of caspase-3. Thus, LA induced cancer cell apoptosis by enhancing cellular oxidant status and inducing mitochondrial dysfunction.

## Introduction

Essential fatty acids (EFAs): linoleic acid (LA, n-6, 18:2) and α-linolenic acid (ALA, n-3, 18:3) form precursors to their long chain metabolites γ-linolenic acid (GLA, n-6, 18:3), dihomo-GLA (DGLA, n-6, 20:3) and arachidonic acid (AA, n-6, 20:4); and eicosapentaenoic acid (EPA, n-3, 20:5) and docosahexaenoic acid (DHA, n-3, 22:6) respectively [1-3]. Our previous studies showed that polyunsaturated fatty acids (PUFAs) selectively induced tumor cells apoptosis though the sensitivity of various cancer cells to different fatty acids were found to be variable depending on the type of cancer cell being tested and the type and concentration of the fatty acid used [3-7].

Previously, it was reported that essential fatty acids and their metabolites suppress tumor cells growth both *in vitro *and *in vivo*. This tumoricidal action of fatty acids could be correlated to an increase in generation of free radicals in the tumor cells [8]. Subsequent studies showed that most polyunsaturated fatty acids were functional, and the inhibitory action of different types of n-3, n-6 and n-9 fatty acids does not depend on their unsaturation [9]. Among all the fatty acids tested, GLA, AA, EPA and DHA were found to be the most effective in inhibiting tumor cells growth, while LA and ALA were also effective but at much higher concentrations [3-5]. It was opined that n-6 fatty acids enhance tumor cell growth whereas n-3 fatty acids are beneficial since they arrest cancer growth. This differential action of n-3 and n-6 PUFAs in cancer has been attributed to the formation of pro-inflammatory eicosanoids from n-6 PUFAs whereas products formed from n-3 PUFAs are much less pro-inflammatory in nature [1-7,10-13], though Trombetta A, *etc*. reported that AA, an n-6 PUFA, decreased human lung-tumor cell growth in a concentration-dependent manner, induction of cell death mainly evident at 100 mM concentration [14].

In the majority of previous investigations, n-3 and n-6 PUFAs were added to the tumor cell medium *in vitro *without a simultaneous study of these fatty acids on normal cells. Hence, it is not clear whether the concentrations of fatty acids used in these studies are non-toxic to normal cells at which they were found to be cytotoxic to tumor cells. In addition, little attention was paid to the ratio between n-6 and n-3 fatty acids as they exist in the body while performing these studies. It is important to note that in the plasma, n-6 PUFAs are present in large amounts compared to n-3 fatty acids (the ratio between n-6 PUFAs compared to n-3 PUFAs is ~7:1 in the serum) [15]. Furthermore, PUFAs are widely distributed in our food and hence, there could be a wide variation in the daily intake of these fatty acids among different populations and individuals depending on the type of diet and the quality of the food ingested. In general, the level of total fatty acids in the plasma/serum is ~200 mg/dl, and of which the percentage of LA is ~20% regardless of the differences in dietary pattern [15].

Previously, we observed that the action of LA on cancer cell growth depended on the type of cancer cells being tested and the concentration of fatty acids supplemented [3-7]. LA ~40 μg/ml/1 × 10^4 ^cells inhibited the growth of cancer cells whereas lower concentrations ~5-10 μg/ml/1 × 10^4 ^cells enhanced growth of some, if not all, types of cancer cells that were being tested [11]. In the present study, we evaluated the effect of the fatty acid on three cell lines, two of which were colorectal cancer cell lines, LOVO (undifferentiated) and RKO (semi-differentiated), and the human umbilical vein endothelial cells (HUVEC) taken as normal cell control in order to clarify the role of LA in the promotion and inhibition of the growth of cancer cells *in vitro*. In order to know the sensitivity of tumor cells to LA, in one set of studies we pre-incubated the cells for 24 hours with 100 μM of LA and then were subsequently exposed to various doses of LA to know whether the initial exposure to LA affects the survival of tumor cells.

## Materials and methods

### Materials

Linoleic acid (LA, 18:2 n-6) was obtained from Sigma (St. Louis, MO, USA). The colorectal cancer cell lines, LOVO (undifferentiated) and RKO (semi-differentiated), and normal cell line HUVEC (human umbilical vein endothelial cells) were obtained from Shanghai Institute of Cell Biology, Chinese Academy of Sciences. PRMI medium 1640 and high-glucose DMEM Nutrient Mixture medium were purchased from GIBCO (Grand Island, NY, USA). MTT (3-(4, 5-dimethylthiazolyl-2)-2, 5-diphenyltetrazolium bromide) was provided by Shanghai Sangon Biological Engineering Technology & Services Co., Ltd. All other chemicals were of extra-pure grade or analytical grade.

### Cell culture and treatment

Colorectal cancer cells (LOVO and RKO) and human normal cells (HUVEC) were cultured in PRMI Medium 1640 and high-glucose DMEM Nutrient Mixture medium separately, supplemented with 10% fetal bovine serum and 100 U/ml penicillin-streptomycin in a humidified 37°C, 5% CO_2 _incubator (Shellab, USA).

LA was dissolved in 0.1 N NaOH and diluted to give a final concentration of 20 mM with the final concentration of NaOH was no more than 0.005 N, a concentration of NaOH at which it had little affect on the cells. Stock solutions were filter-sterilized and diluted with cell culture media for use in the study [16].

Both the colon cancer cells (LOVO and RKO) and normal cell (HUVEC) were treated with LA in two different ways: in the first group, the cells were cultured in the cell culture medium alone for 24 h prior to treatment with different doses of LA; whereas in the second group, cells were pre-incubated with 100 μM LA for 24 hr followed by treatment with different doses of LA as was done in the first group.

### Cell growth and viability assay

Cell proliferation was assessed using MTT assay (Roche, Mannheim, Germany). At different time intervals after incubation with LA, the number of viable cells grown in a 96-well plate was estimated by adding 20 μl of MTT solution (5 mg/ml in PBS). After 4 hr of incubation at 37°C, the stain was diluted with 150 μl of DMSO. The absorbance in each well was then measured with a microplate reader (Thermal Lab system, Finland) at 492 nm, and viability of cells was presented as percentage of the control [16]. Each treatment was replicated at least five times.

### Mitochondrial membrane potential detection

Cells (1 × 10^6 ^cells) obtained from control and various LA treatments were washed thrice with PBS, and resuspended at a final protein concentration of 0.75 mg/ml in PBS, 100 μl was taken for the following detection. 100 ng Rh123 (Rhodamine 123) were added to each sample. Upon incubation in the dark (15 min, at room temperature or 30 min at 4°C), the samples were washed with 300 μl PBS twice. The cells were resuspended in 200 μl PBS for analysis. Fluorescence intensity was carried out on a multifunctional micro-plate reader (SpectraMax M5, Molecular Devices) with a 96-well plate (side-opaque, clear bottom). The excitation and emission wavelengths for Rh123 were selected with monochromators set to 488 nm (5 nm slit width) and 530 nm (5 nm slit width), respectively. Each treatment was replicated thrice, and the final data was calculated as follows: corresponding fluorescence intensity (%) = F_sample_/F_CK_

Where: F_sample _and F_CK _are the intensities measured with microplate reader. [17]

### **ROS generation studies**

Cells were incubated with the cell permeant dye H_2_DCF-DA, which intracellularly de-esterifies to dichlorodihydrofluorescein (H_2_DCF). ROS oxidize H_2_DCF to the brightly fluorescent compound 2-, 7-dichlorofluorescein (DCF), which was monitored by flow cytometry following a previously described method [18] with modifications. Briefly, cells treated with LA or vehicle were incubated with 5 μM H_2_DCF-DA for 15 min at 37°C, detached from the plate with trypsin/EDTA, washed with PBS, resuspended in ice-cold PBS, and tested immediately. Triplicate samples were run in each experiment, and at least 5000 cells per sample were analyzed (excitation at 488 nm, emission at 500-530 nm) by *SpectraMax M5, Molecular Devices*. Mean fluorescence was calculated by using the program *SoftMax Pro Software Version 5 for Mac^® ^and Windows^®^*. The final date was calculated as given below:

Corresponding fluorescence intensity (%) = F_sample_/F_CK_

Where: F_sample _and F_CK _are the intensities measured with microplate reader.

### Estimation of cell MDA content and SOD activity

The levels of MDA and the activity of SOD, the biomarkers of oxidative stress, were measured as described previously with commercial reagent kits purchased from Nanjing Kaiji Bioengineering Institute (Nanjing, China) [19]. The cell MDA content and SOD activity were also represented as corresponding value:

Corresponding MDA (or SOD) concentration (%) = C_sample_/C_CK_

Where: C_sample _and C_CK _are the concentration of MDA (or SOD) measured with regent kits.

### Isolation and purification of mitochondria

The cells were harvested by centrifuging at 1,500 rpm for 5 min at 4°C, and washed twice with cold PBS and finally re-suspended in PBS. Cells were lysed by Ultrasonic Cell Disruption System (JY92-II, Chongqing, China) and centrifuged at 3,000 rpm for 10 min at 4°C. The supernatant thus obtained was centrifuged at 9,000 rpm for 10 min to obtain mitochondrial pellets that were washed twice with cold PBS. The final mitochondrial pellets were suspended in test medium (220 mmol/L Mannitol, 70 mmol/L Sucrose, 5 mmol/L HEPES, pH 7.2) for the following studies. The final protein concentrations of the mitochondrial suspension were all adjusted to 0.3 mg/mL (detected and corrected with Bradford protein analysis kit, Sangon, Shanghai, China) [20].

### Measurement of cytochrome C content

Isolated mitochondrial content of cytochrome C was measured at 520 nm in UV-vis spectroscopy (HP 8453, Hewlett-Packard, USA) by suspending them in a reaction medium containing 10 mg sodium dithionite and 0.5 mL of 0.3 mg/mL mitochondrial protein. Sample concentrations were determined based on a standard curve. Each of the experiments was replicated three times [20].

### Measurement of the activities of caspases 9 and 3

The activities of caspases 9 and 3 were assayed using caspase-9 and caspase-3 activity assay kits separately, according to the manufacture's instructions (Keygen, Nanjing, China). After different treatments, 5 × 10^6 ^cells were harvested by centrifuging at 1,500 rpm for 5 min at 4°C, and washing twice with cold PBS, then resuspended in a cell lysis buffer. After incubation on ice for 60 min, the lysates were centrifuged for 20 min at 12,000 rpm, the supernatants were collected and protein concentrations were determined. Cell lysates (100 μg) were mixed with reaction buffer and incubated for 4 h at 37°C. The absorbance of the reaction mixture was measured in the wells at 405 nm using an ELISA reader (Thermal Labsystem, Helsinki, Finland) [20].

### Statistical analysis

All results are expressed as means ± SD and significance of differences between various results was determined by Origin 7.0. Each treatment was carried out at least triple, and statistic analysis was shown as: * meaning p < 0.05, and for ** meaning p < 0.01.

## Results

### Cell growth and viability

**Figure 1:**
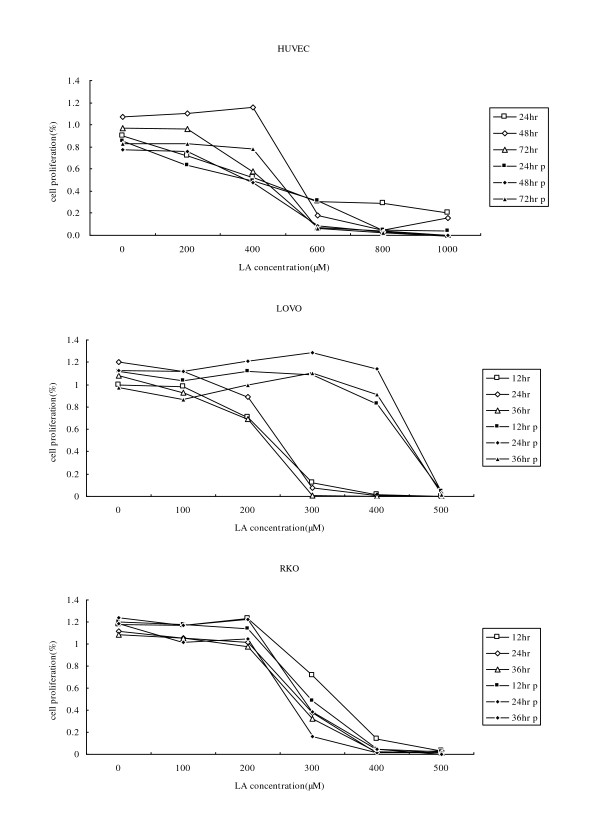
**MTT assay results**. The three figures are the results of the cell proliferation assay of HUVEC, LOVO, and RKO cell lines separately. In each figure, "12 h, 24 h, 36 h" means cells were cultured with gradient LA treatment after 12 hour, 24 hour, 36 hour with no linoleic acid medium and incubated for 24 h; "12 h p, 24 h p, 36 h p" means cells were cultured with 100 μM LA for 24 hr before gradient LA treatment. Each treatment was carried out at least five times.

All the three cell lines studied showed increased cell proliferation at lower concentrations of LA (100-200 μM), while higher concentrations of LA (above 300 μM) suppressed the growth of the cells as determined by MTT assay (Fig. 1).

It is interesting to note that different cell lines showed different sensitivity to LA depending on the type of cell line being tested and the dose of LA supplemented (Fig. 1). Of three types of cells tested, HUVEC were the most resistant to the cytotoxic action of LA compared to the two cancer cell lines used in the present study. The colorectal cancer cell lines showed increased or no change in proliferation when exposed to 100 μM LA whereas HUVEC cells remained non-responsive even when supplemented with 400 μM LA. LA suppressed the proliferation of LOVO and RKO cells to a significant degree when supplemented with 300 μM of LA, whereas HUVEC cells showed decreased proliferation only when incubated with 600 μM of LA. Based on these results, we chose 200 μM and 400 μM for treatment of HUVEC, while 100 μM and 300 μM LA was used to study the effects of LA on colorectal cancer cells for subsequent studies.

On the other hand, when we studied the effect of pre-incubation of cells with 100 μM of LA for 24 hours followed by further exposure to various doses of LA, it was noted that the sensitivity of HUVEC to the growth suppressive action of LA was enhanced. For instance, it was noted that almost 50% of HUVEC were still surviving even when treated with 400 μM LA after pre-treatment. Of the two cancer cell lines studied, LOVO cells were found to be resistant to the growth suppressive actions of LA even when exposed to 300 μM LA after pre-treatment, while, RKO cells showed increased sensitivity to the growth suppressive action of the fatty acid. Hence, we performed all subsequent mechanistic studies with HUVEC, LOVO and RKO cells by pre-incubating them with 100 μM for 24 hours followed by subsequent supplementation with LA as described.

### Cell mitochondrial membrane potential detection

**Figure 2:**
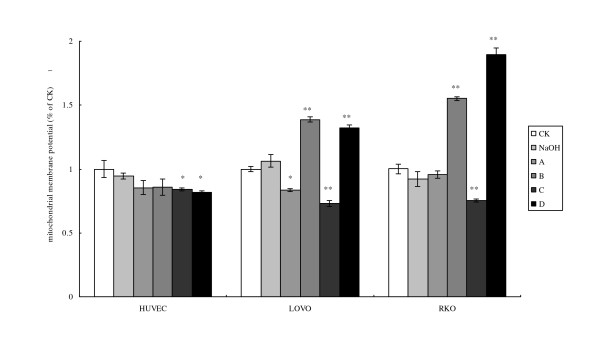
**Cell mitochondrial membrane potential detection**. CK means case-control with medium. As LA was dissolved in 0.1 N NaOH, NaOH group means case-control with reagent. A, B, C, and D were four different treatments of each cell line. **For HUVEC: **A = 200 μM without LA pre-treatment, B = 400 μM without LA pre-treatment, C = 200 μM with 100 μM LA pre-treatment, D = 400 μM with 100 μM LA pre-incubation, **For LOVO and RKO cells: **A = 100 μM without LA pre-treatment, B = 300 μM without LA pre-treatment, C = 100 μM with 100 μM LA pre-treatment, D = 300 μM with 100 μM LA pre-incubation. Each treatment was carried out in triplicate and repeated at least twice. *p < 0.05, and **p < 0.01.

Cell mitochondrial membrane potential was evaluated as a measure of the degree of injury to the mitochondrial membrane which is reflected in the amount of permeability exhibited to Rh123 (Fig. 2): the higher the permeability the higher fluorescence of Rh123 could pass-through mitochondrial membrane indicating statically significant damage to the membrane.

HUVEC did not show any significant change in the cell mitochondrial membrane potential following the addition of LA, while the two cancer cell lines LOVO and RKO showed significant yet similar changes in the mitochondrial membrane potential in response to incubation with various treatments with LA. Higher concentration of LA (300 μM) with and without pre-treatment enhanced mitochondrial uptake of Rh123 by LOVO and RKO cells compared with their respective controls, while the low concentration treatments (100 μM of LA) did not show any significant change in mitochondrial membrane fluorescence permeability of Rh123 (Fig. 2). It is noteworthy that both LOVO and RKO cells showed similar increase in the mitochondrial membrane fluorescence permeability of Rh123 in response to incubation with 300 μM of LA with or without pre-incubation (Fig. 2).

Pre-incubation with LA increased fluorescence intensity of LOVO and RKO cells, but not of HUVEC, suggesting that HUVEC are able to maintain their mitochondrial function while the colon cancer cells could not. In addition, pre-incubation of RKO cells with LA (100 μM) and subsequent exposure to 300 μM of LA led to a much higher fluorescence intensity of Rh123 in comparison to LOVO cells. The higher fluorescence intensity of Rh123 by RKO cells, the semi-differentiated cells, in comparison to the fluorescence intensity by LOVO cells, the undifferentiated cells, in response to the same dose of LA could be related to the cell differentiation state. The higher fluorescence intensity of Rh123 by LA-treated RKO cells in comparison to fluorescence intensity shown by LOVO cells indicates that LA is able to induce much higher damage to the mitochondrial membrane of RKO cells compared to the LOVO cells. These results indicate that RKO cells are much more sensitive to the toxic actions of LA compared to LOVO cells.

### Cell oxidative stress analysis

In order to know the degree of oxidative stress that occurred as a result of incubation with LA, we studied generation of ROS, MDA accumulation, and SOD activity in the cells.

**Figure 3:**
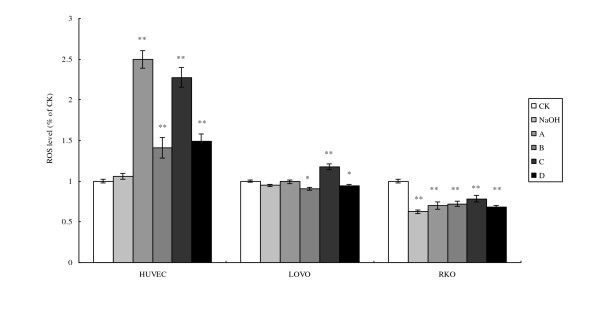
**Cell ROS (reactive oxygen species) level**. CK means case-control with medium. As LA was dissolved in 0.1 N NaOH, NaOH group means case-control with reagent. A, B, C, and D were four different treatments of the three cell lines. **For HUVEC: **A = 200 μM without LA pre-treatment, B = 400 μM without LA pre-treatment, C = 200 μM with 100 μM LA pre-treatment, D = 400 μM with 100 μM LA pre-incubation. **For LOVO and RKO cells: **A = 100 μM without LA pre-treatment, B = 300 μM without LA pre-treatment, C = 100 μM with 100 μM LA pre-treatment, D = 300 μM with 100 μM LA pre-incubation. Each treatment was carried out in triplicate. *p < 0.05, and **p < 0.01.

The amount of ROS generated by HUVEC when incubated with 200 μM LA with and without pre-incubation was at least 2 to 2.5 times higher compared to the untreated control while exposure to 400 μM of LA produced much less (only about 50% rise) increase (Fig. 3). In contrast, both the colorectal cancer cell lines RKO and LOVO studied showed much less significant change in the generation of ROS in response at both doses of LA (100 and 300 μM) added. There was no significant difference in the amount of ROS generated with or without pre-incubation with 100 μM of LA in colorectal cancer cell lines.

**Figure 4:**
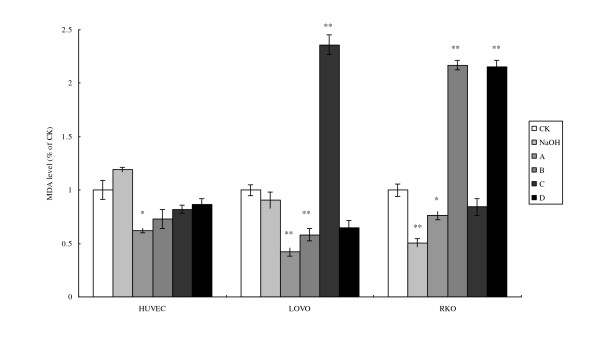
**Cell MDA content**. CK means case-control with medium. As LA was dissolved in 0.1 N NaOH, NaOH group means case-control with reagent. A, B, C, and D were four different treatments of the three cell lines. **For HUVEC: **A = 200 μM without LA pre-treatment, B = 400 μM without LA pre-treatment, C = 200 μM with 100 μM LA pre-treatment, D = 400 μM with 100 μM LA pre-incubation, **For LOVO and RKO cells: **A = 100 μM without LA pre-treatment, B = 300 μM without LA pre-treatment, C = 100 μM with 100 μM LA pre-treatment, D = 300 μM with 100 μM LA pre-incubation. Each treatment was carried out in triplicate and repeated at least twice. *p < 0.05, and **p < 0.01.

In contrast to the results obtained with regard to ROS generation, the accumulation of MDA in the cells showed distinctly different results. There were no significant changes in the levels of MDA accumulation in HUVEC following incubation with different doses of LA (Fig. 4), while colorectal cancer cells LOVO and RKO showed about 2 ~2.5 times increase compared to respective controls. Pre-incubation with LA (100 μM) significantly enhanced MDA accumulation in LOVO, while RKO cells showed enhancement in the MDA levels upon treatment with LA with and without pre-incubation with 100 μM of LA compared to the respective controls (Fig. 4).

**Figure 5:**
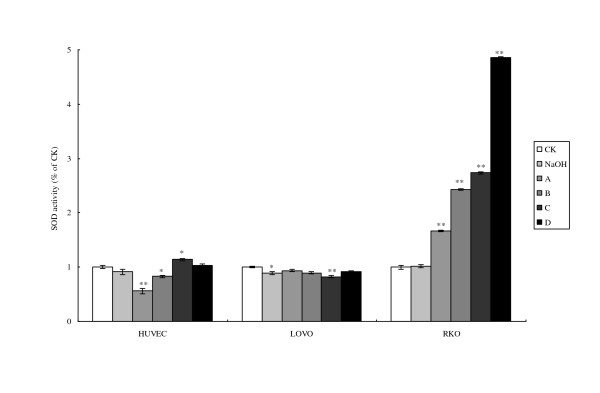
**Cell SOD activity**. CK means case-control with medium. As LA was dissolved in 0.1 N NaOH, NaOH group means case-control with reagent. A, B, C, and D were four different treatments of the three cell lines. **For HUVEC: **A = 200 μM without LA pre-treatment, B = 400 μM without LA pre-treatment, C = 200 μM with 100 μM LA pre-treatment, D = 400 μM with 100 μM LA pre-incubation, **For LOVO and RKO cells: **A = 100 μM without LA pre-treatment, B = 300 μM without LA pre-treatment, C = 100 μM with 100 μM LA pre-treatment, D = 300 μM with 100 μM LA pre-incubation. Each treatment was carried out in triplicate and repeated at least twice. *p < 0.05, and **p < 0.01.

LA treatment raised SOD activity in RKO cells to a significant degree in a time and dose dependent fashion (Fig. 5), while the there were no significant changes in SOD activity in HUVEC and LOVO cells.

### Cell mitochondrial apoptosis pathway analysis

**Figure 6:**
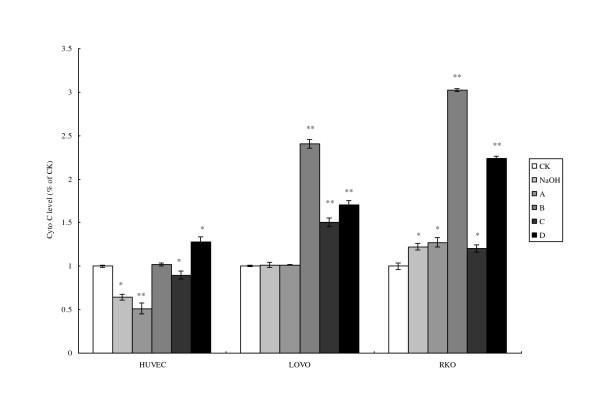
**Cell cytochrome C concentration**. CK means case-control with medium. As the LA was dissolved in 0.1 N NaOH, NaOH group means case-control with reagent. A, B, C, and D were four different treatments of the three cell lines. **For HUVEC: **A = 200 μM without LA pre-treatment, B = 400 μM without LA pre-treatment, C = 200 μM with 100 μM LA pre-treatment, D = 400 μM with 100 μM LA pre-incubation. **For LOVO and RKO cells: **A = 100 μM without LA pre-treatment, B = 300 μM without LA pre-treatment, C = 100 μM with 100 μM LA pre-treatment, D = 300 μM with 100 μM LA pre-incubation. Each treatment was carried out in triplicate and repeated at least twice. *p < 0.05, and **p < 0.01.

Cytochrome C release induces mitochondrial dependent apoptosis. An enhanced release of cytochrome C was noted in HUVEC treated with LA with and without pre-incubation with LA (100 μM) (Fig. 6). An increased release of cytochrome C was noted in both LOVO and RKO cells treated with LA with or without pre-treatment. It is noteworthy that the release of cytochrome C was much higher in cells that were treated with higher dose of LA (300 μM) with or without pre-incubation with LA (Fig. 6).

**Figure 7:**
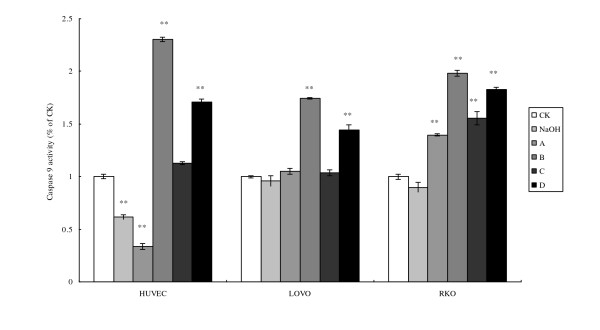
**Cell caspase-9 activity**. CK means case-control with medium. As LA was dissolved in 0.1 N NaOH, NaOH group means case-control with reagent. A, B, C, and D were four different treatments of the three cell lines. **For HUVEC: **A = 200 μM without LA pre-treatment, B = 400 μM without LA pre-treatment, C = 200 μM with 100 μM LA pre-treatment, D = 400 μM with 100 μM LA pre-incubation, **For LOVO and RKO cells: **A = 100 μM without LA pre-treatment, B = 300 μM without LA pre-treatment, C = 100 μM with 100 μM LA pre-treatment, D = 300 μM with 100 μM LA pre-incubation. Each treatment was carried out in triplicate and repeated at least twice. *p < 0.05, and **p < 0.01.

**Figure 8:**
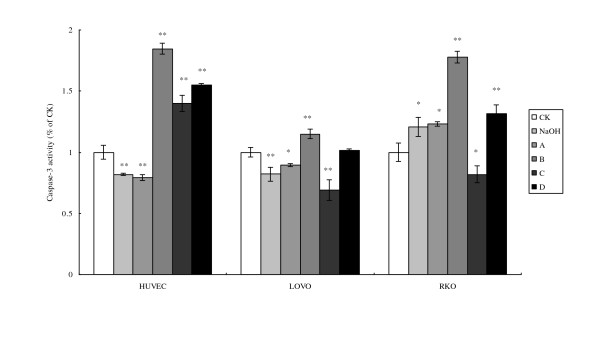
**Cell caspase-3 activity**. CK means case-control with medium. As LA was dissolved in 0.1 N NaOH, NaOH group means case-control with reagent. A, B, C, and D were four different treatments of the three cell lines. **For HUVEC: **A = 200 μM without LA pre-treatment, B = 400 μM without LA pre-treatment, C = 200 μM with 100 μM LA pre-treatment, D = 400 μM with 100 μM LA pre-incubation, **For LOVO and RKO cells: **A = 100 μM without LA pre-treatment, B = 300 μM without LA pre-treatment, C = 100 μM with 100 μM LA pre-treatment, D = 300 μM with 100 μM LA pre-incubation. Each treatment was carried out in triplicate and repeated at least twice. *p < 0.05, and **p < 0.01.

Enhanced release of cytochrome C activates caspase family that induces cell apoptosis. As expected, the changes in the activation of caspases 9 and 3 paralleled the changes seen with cytochrome C release in HUVEC, LOVO and RKO cells (Fig. 7 and 8). RKO cells showed much higher increases in the activation of caspase 3 compared to caspase 9, while LOVO cells showed much less activation of caspase 3 compared to caspase 9.

## Discussion

N-6 PUFAs are known for their critical role in many physiological functions but their role in cancer is still under debate. Both tumor promoting and inhibiting properties of n-6 fatty acids have been reported. It has been shown that n-6 fatty acids decrease human lung-tumor cell growth in a concentration-dependent manner [21,14]. Our results suggested that low concentrations (≤ 200 μM) of LA promote colorectal cancer cell growth, while high levels (≥ 200 μM) induce apoptosis of the colorectal cancer cells *in vitro*. On the other hand, low concentrations of LA (≤ 200 μM) did not promote normal (HUVEC) cell proliferation while high concentrations (≥ 200 μM), which were cytotoxic to tumor cells, induced only 10~20% decrease in the number of HUVEC. These results suggest that LA is toxic to tumor cells with little or no cytotoxic action on normal cells.

Mitochondria are the main organelles where fatty acids and other energy donors are metabolized/oxygenized to support cell survival. In the present study, it was noted that the mitochondrial membrane potential is affected only when cells were exposed to ≥ 200 μM of LA, while low LA concentrations (≥ 200 μM) were without any affective. It was also observed that pre-treatment with LA followed by supplementation with high concentrations of LA induced significant increase in fluorescence suggesting that mitochondrial membrane loses its ability to function as an electron transporter and aerobic respirator (Fig. 2) at the dose tested.

PUFAs enhance free radical generation [22] that may be related to the loss of mitochondrial function. Previously, it was reported that tumoricidal action of PUFAs could be correlated to an increase in generation of free radicals [8]. We also noted that GLA induced apoptosis of tumor cells by augmenting free radical generation only in the tumor cells but not normal cells [23]. Based on the results of the present study and previous results [24], we suggest that free radical generation is the main mediator of mitochondrial damage and apoptosis of tumor cells. Despite similar treatment schedules, the formation of ROS, lipid peroxides and the degree of apoptosis were found to be dissimilar between LOVO and RKO cells. It is noteworthy that the levels of SOD were significantly elevated in RKO compared with LOVO cells suggesting that oxidant stress is present to a significant degree in the former but not the latter. The increase in SOD levels in RKO could be secondary to the accumulation of significant amounts of MDA in these cells. The formation of ROS and lipid peroxides were not significantly increased in HUVEC suggesting that normal cells resist the formation of lipid peroxides and generation of ROS and thus are able to with stand oxidant stress induced by exposure to LA (Figs. 3, 4, &5).

MDA formulation is mainly due to (a) non-enzymatic free radical catalyzed lipid peroxidation and (b) AA derived cyclooxygenase pathway that can be enhanced by the addition of LA. Pre-treatment with LA (100 μM) followed by supplementation of 100 and 300 μM of LA enhanced the formation of lipid peroxides (MDA formation) in RKO cells compared with HUVEC and LOVO cells suggesting that accumulation or formation of lipid peroxides in cells may vary depending on the cell type. These results imply that accumulation of lipid peroxides may trigger apoptosis and generation of ROS and the levels of SOD could be determined by the amount and degree of lipid peroxides formed in the cells.

**Figure 9:**
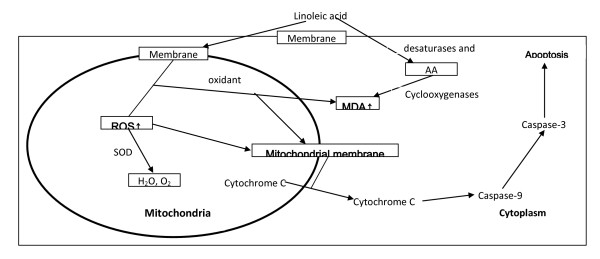
**Mitochondrial mediate apoptosis pathway**.

Mitochondrial dysfunction leads to apoptosis due to the release of cytochrome C that, in turn, activates caspase-9 and caspase-3. Oxidant stress can induce mitochondrial dysfunction, alter its membrane potential, interrupt electron transport chain and induce apoptosis. LA-treated tumor cells showed significantly enhanced cytochrome C release, and activities of caspase-9 and caspase-3 (Figs. 7 and 8). Enhanced activity of caspase-9 and cytochrome c release was observed in LA-pre-treated RKO tumor cells that support the proposal that apoptosis in these cells are mediated by mitochondrial pathway (Figure 9). These results suggest that accumulation of LA in the tumor cells could facilitate apoptotic process since LA-pre-treated tumor cells were found to be much more vulnerable to apoptosis compared to untreated tumor and normal cells.

Cell differentiation status could affect their sensitivity to the cytotoxic actions of LA. This is supported by the observation that the differentiated cell line RKO was more sensitive to the cytotoxic action of LA in comparison to the undifferentiated cell line LOVO, especially after pre-incubation with LA. These results imply that LOVO cells may metabolize LA more efficiently and are able to detoxify the toxic metabolites of LA in comparison to RKO cells. Hence, it will be interesting to study the differential metabolic pathway of LA in RKO, LOVO cells and HUVEC as related to its cytotoxic action.

Although the results of the present study indicated that colorectal cancer cell lines are more susceptible to the cytotoxic effects of LA compared with human umbilical endothelial cells, the inherent cell type differences may also have influenced the differential respones observed rather than tumor specific differences. Hence, more studies need to be performed to understand and elucidate the molecular mechanism(s) of the cytotoxic action of LA.

In summary, it was observed that LA induces the formation of lipid peroxides that, in turn, could induce apoptosis of tumor cells. The differential cytotoxicity observed in two different colorectal cancer cell lines at different stages of differentiation suggests that the handling of LA by these cells is different and understanding its (LA) metabolism in these cells may lead to the identification toxic metabolites formed that drive its cytotoxic action.

## Abbreviations used

AA: arachidonic acid; ALA: α-linolenic acid; DGLA: dihomo-γ-linolenic acid; DHA: docosahexaenoic acid; EPA: eicosapentaenoic acid; GLA: γ-linolenic acid; LA: linoleic acid; MDA: malondialdehyde; mPTP: mitochondrial permeability transition pore; MTT: 3-(4, 5-dimethylthiazolyl-2)-2, 5-diphenyltetrazolium bromide; Rh123: Rhodamine 123; ROS: reactive oxygen species; SOD: Superoxide Dismutase.

## Declaration of competing interests

The authors declare that they have no competing interests.

## Authors' contributions

SS and UND conceived the idea and designed the experiments. XL, HY and QM performed the experiments. SS participated in the design of the study and performed the statistical analysis. SS and UND drafted the manuscript and interpretation of the data. All authors read and approved the final manuscript.
